# Five-Year Long-Term Results of Standard Collagen Cross-Linking Therapy in Patients with Keratoconus

**DOI:** 10.4274/tjo.galenos.2020.53810

**Published:** 2020-08-26

**Authors:** Yelda Yıldız Taşçı, Ayşe Güzin Taşlıpınar Uzel, Demet Eyidoğan, Özge Saraç, Nurullah Çağıl

**Affiliations:** 1 Ankara City Hospital, Clinic of Ophthalmology, Ankara, Turkey; 2 Sandıklı State Hospital, Clinic of Ophthalmology, Afyon, Turkey; 3 Midyat State Hospital, Clinic of Ophthalmology, Mardin, Turkey; 4 Ankara Yıldırım Beyazıt University Faculty of Medicine, Department of Ophthalmology, Ankara, Turkey

**Keywords:** Keratoconus, collagen cross-linking, standard Dresden protocol

## Abstract

**Objectives:**

We aimed to demonstrate the 5-year visual, topographic, and aberrometry long-term results of standard collagen cross-linking (CCL) treatment in keratoconus patients.

**Materials and Methods:**

The files and topographic measurements of patients who underwent standard CCL treatment for progressive keratoconus were retrospectively reviewed. Uncorrected visual acuity (UCVA), best corrected visual acuity (BCVA), refraction values, and topographic values were evaluated.

**Results:**

Thirty-seven eyes of 27 patients were included in the study. The female to male ratio was 15 (56%)/12 (44%) and the mean age was 22.16±6.4 (12-39) years. The increase in UCVA and BCVA was statistically significant at postoperative 1-5 years (all p values <0.05). The changes in the spherical equivalent after CCL were not statistically significant (p>0.05), but the decrease in the manifest astigmatism values were significant after CCL at 3-5 years (p<0.05). Decrease in K2 (steep keratometry) and K apex values were statistically significant at 1-5 years (p<0.05). There was a significant decrease in the thinnest corneal thickness compared to the preoperative values up to 6 months and 1-4 years (p<0.05), but the change at 5 years was not significant (p=0.08). Post-CCL reductions in high-order aberrations and spherical aberrations were significant at postoperative 5 years and 3-5 years (p<0.05).

**Conclusion:**

In long-term follow-up, CCL treatment is seen to arrest keratoconus progression, increase vision, and improve visual quality by reducing higher-order aberrations and spherical aberrations. For these reasons, CCL treatment continues to be the first treatment modality in patients with progressive keratoconus.

## Introduction

Keratoconus is a progressive corneal ectasia characterized by thinning and protrusion of the cornea. It is usually bilateral and may be asymmetric or symmetric.^1,2^ Its prevalence is 1/2000 and it affects both sexes equally.^1^ Although its etiology has not been fully elucidated, environmental, genetic, and inflammatory factors are believed to be responsible.^3,4,5,6^

Studies on both the pathology and pathogenesis of keratoconus have demonstrated reduced collagen cross-links, decreased collagen fiber diameter, and weaker enzymatic resistance.^7^ Treatment involves glasses, contact lenses, corneal collagen cross-linking (CCL), intracorneal ring, and in more severe cases, lamellar or penetrating keratoplasty. In 2003, Wollensak et al.^8^ showed that applying riboflavin and ultraviolet A (UVA) could increase corneal cross-linking through additional chemical bonds, thereby enhancing the mechanical strength of the cornea and halting the progression of keratoconus. CCL, which is the first-line treatment for keratoconus, is currently the only treatment method that halts progression and reduces future need for corneal transplantation.

The aim of our study was to show the 5-year visual, topographical, and aberrometric outcomes of standard therapeutic CCL in keratoconus patients.

## Materials and Methods

This retrospective study was performed in the Keratoconus Center of the Ankara Atatürk Training and Research Hospital and adhered to the principles of the Declaration of Helsinki. Ethics committee approval for the study was obtained from the Clinical Research Ethics Committee of the Yıldırım Beyazıt University Faculty of Medicine. The records and topographical measurements of patients who underwent standard CCL due to progressive keratoconus and attended regular follow-up visits in our center between 2012 and 2018 were retrospectively evaluated. All keratoconus patients younger than 18 years of age were included in the study regardless of progression; for patients over 18 years of age, only those with progression were included. An increase greater than 1 D in K apex value within the last year was considered keratoconus progression. Patients with corneal scar, history of herpetic keratitis or ocular surgery, thinnest corneal thickness (TCT) less than 370 µm, and those who did not attend regular follow-up examinations were not included in the study.

All patients included in the study underwent CCL with the standard Dresden Protocol. The surgical technique involved the application of topical 0.5% proparacaine hydrochloride (Alcaine, Novartis, Alcon-Couvreur, Belgium) or general anesthesia, after which an 8.5-mm diameter flap of corneal epithelium was lifted mechanically and 0.1% hypotonic riboflavin solution (Merribo hypoosmolar, Meran Medical, İstanbul, Turkey) was instilled on the cornea at 1-minute intervals for 30 minutes. When cornea thickness exceeded 400 µm, 3 mW/cm^2^ UVA (Meran Medical, BNM, İstanbul) was applied for 30 minutes, followed by placement of a bandage contact lens. For the first postoperative week, the patients were prescribed fluorometholone 0.1% (Flarex 0.1% ophthalmic solution, Novartis, Alcon-Couvreur, Belgium)  4 times a day, 0.3% ofloxacin (Exocin ophthalmic solution 0.3%, Allergan, Ireland) 4 times a day, preservative-free polyvinyl alcohol + povidone (Refresh, Allergan, Ireland) 6 times a day. Fluorometholone therapy was tapered and discontinued after 2 months. The patients’ preoperative data and follow-up data from postoperative month 6 and years 1-5 were evaluated. Analyzed parameters included uncorrected visual acuity (UCVA), best corrected visual acuity (BCVA), manifest cylindrical value and spherical equivalent, presence of corneal haze, and the following topographic data (Sirius Scheimpflug Camera, Italy): K1 (flat keratometry), K2 (steep keratometry), K apex, topographic corneal astigmatism, TCT, and corneal aberrations (root mean square [RMS] representing the standard deviation of total aberrations, RMS of higher-order aberrations (HORMS), vertical trefoil, vertical coma, horizontal coma, oblique trefoil, oblique quadrafoil, oblique secondary astigmatism, spherical aberration, vertical secondary astigmatism, and vertical quadrafoil).

### Statistical Analysis

Data were recorded and analyzed using IBM SPSS Statistics version 21.0 (IBM Corp, Armonk, NY) and MedCalc version 12.3 (MedCalc Software bvba, Ostend, Belgium). Normality of data distributions were analyzed using Kolmogorov-Smirnov test. Mean and standard deviations were calculated. For repeated measures, paired samples t-test was used for numerical values and marginal homogeneity test for categorical variables. Analyses were performed with a 95% confidence interval and a p value less than 0.05 was considered statistically significant.

## Results

**Figure 1:**
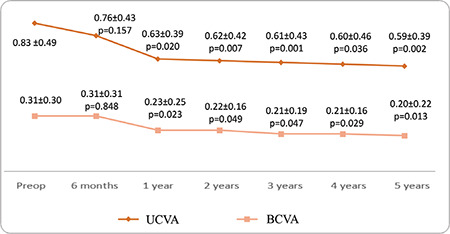
Mean uncorrected visual acuity (UCVA) and best corrected visual acuity (BCVA) according to preoperative (preop) and postoperative logMAR

**Figure 2:**
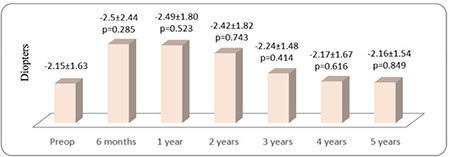
The patients’ preoperative (preop) and postoperative mean spherical equivalent values

**Figure 3:**
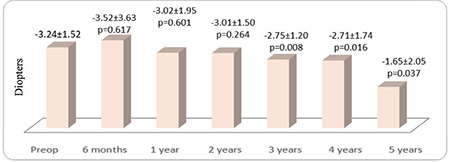
The patients’ preoperative (preop) and postoperative mean manifest astigmatism values

Thirty-seven eyes of 27 patients were included in the study. The female to male ratio was 15/12 (56%/44%) and the mean age was 22.16±6.4 (12-39) years. The patients’ preoperative and postoperative 6-month and 1- to 5-year mean UCVA and BCVA values according to logMAR are shown in Figure 1. UCVA and BCVA values before CCL and at 6 months and 1, 2, 3, 4, and 5 years after CCL were parallel (Figure 1). After postoperative month 6, there were significant improvements at year 1, 2, 3, 4, and 5 in both UCVA (p=0.020, 0.007, 0.001, 0.036, and 0.002, respectively) and BCVA (p=0.023, 0.049, 0.047, 0.029, and 0.013, respectively) (Figure 1). Mean visual improvement on Snellen chart was 0.8, 0.5, 0.7, 0.7, and 0.9 lines at 1, 2, 3, 4, and 5 years after treatment, respectively. None of the patients had 1 or more lines of vision loss. Rates of grade 0, 1, 2, and 3 corneal haze after CCL were 64%, 30%, 6%, 0% at 6 months; 61%, 36%, 3%, 0% at 1 year; 78%, 18%, 4%, 0% at 2 years; 85%, 15%, 0%, 0% at 3 years; 81%, 19%, 0%, 0% at 4 years; and 87%, 13%, 0%, 0% at 5 years, respectively. The decrease in corneal haze was statistically significant after year 2 (p<0.05). The patients’ mean spherical equivalent and manifest astigmatism values preoperatively and at postoperative month 6 and years 1-5 are shown in Figures 2 and 3, respectively. There were no significant changes in spherical equivalent after CCL (p>0.05, Figure 2), whereas manifest astigmatism values were significantly lower at postoperative year 3, 4, and 5 (p<0.05, Figure 3).

**Figure 4:**
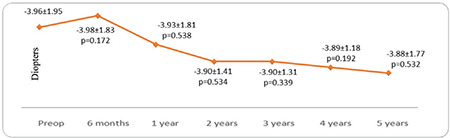
The patients’ preoperative (preop) and postoperative topographic corneal astigmatism values

**Figure 5:**
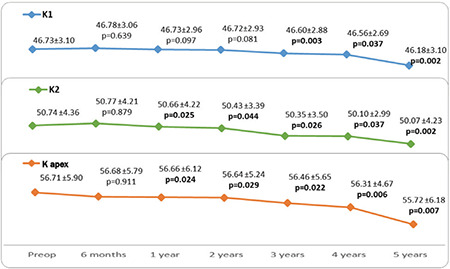
The patients’ preoperative (preop) and postoperative mean K1 (flat keratometry value), K2 (steep keratometry value), and K apex values

**Figure 6:**
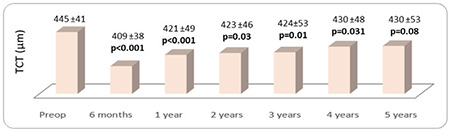
The patients’ preoperative and postoperative mean thinnest corneal thickness (TCT) values

No significant change was observed in topographic corneal astigmatism values after CCL (p>0.05, Figure 4). The patients’ mean pre- and postoperative K1, K2, and K apex values are shown in Figure 5. K1 decreased significantly at postoperative year 3, 4, and 5 (p=0.003, 0.037, and 0.002, respectively, Figure 5). The significant decrease in K2 and K apex values starting at postoperative year 1 persisted at year 2, 3, 4, and 5 (p<0.05 for all, Figure 5). The mean pre- and postoperative TCT values are presented in Figure 6. A significant decrease in TCT compared to preoperative values was observed at postoperative month 6 and persisted at years 1-4 (p=0.001, 0.001, 0.03, 0.01, and 0.031, respectively, Figure 6). No significant change was observed between preoperative TCT and TCT at postoperative year 5 (p=0.08, Figure 6). Progression was observed in 2 eyes of 2 patients (5.4%). These patients underwent a second CCL procedure following the same protocol and showed no further progression in subsequent follow-ups. In post-treatment corneal aberration parameters, no significant changes were observed in RMS, vertical trefoil, vertical coma, horizontal coma, oblique trefoil, oblique quadrafoil, oblique secondary astigmatism, vertical secondary astigmatism, or vertical quadrafoil (p>0.05), while the decreases in HORMS at postoperative year 5 and spherical aberration at postoperative years 3-5 were statistically significant (p<0.05, Table 1). Although no significant correlations were detected between HORMS and BCVA at year 5 (p=0.096, r=0.278), there was a significant negative correlation between spherical aberration and BCVA (p=0.009, r=-0.423).

## Discussion

Keratoconus is an important corneal disease that is more prevalent in young patients and can progress to cause severe visual impairment if left untreated. When a sufficient level of vision cannot be attained in eyes with keratoconus using glasses or contact lenses, the patient may end up undergoing corneal transplantation surgery, which poses the risk of severe complications. Therefore, the early detection of keratoconus and timely application of CCL, a treatment which arrests the disease, are highly important. CCL treatment enhances the biomechanical strength of the cornea and prevents keratoconus progression.^9^ In the international literature, there are few studies reporting the outcomes of CCL treatment after 5 or more years.^10,11,12^ Studies conducted in Turkey show 2-year outcomes at most.^13,14^ Thus, ours is the first study in our country to report the 5-year outcomes of CCL.

In the present study, we found that UCVA and BCVA were stable in the first 6 months and increased at 1, 2, 3, 4, and 5 years after CCL. While several studies have shown consistent findings of increased BCVA after CCL,^13,15,16^ there are other studies in which vision level did not change after CCL.^17,18^ In our study, changes in mean spherical equivalent values after CCL were not significant, but the decrease in manifest astigmatism values was significant. Consistent with our results, there are studies showing no change in spherical equivalent values after CCL,^19,20,21^ but there are also studies showing a decrease in spherical equivalent values after CCL.^22,23,24^ There are now also many studies demonstrating reduced manifest astigmatism values after CCL treatment performed using the epi-off method.^15,25,26,27^ The results of our study further support evidence that CCL performed using the epi-off method flattens the corneal surface and improves cylindrical refraction value. Refraction examination of keratoconus patients shows high variability, and it can sometimes be difficult to determine a patient’s refraction values and level of vision. In such cases, subjective refraction examination can be useful. Patients scheduled to undergo CCL (or the legal guardians of minor patients) must be informed that vision may improve after treatment, but may also remain the same or be further impaired due to potential complications of the procedure.

In our study, the change in corneal astigmatism values after CCL was not significant. We attribute this to the similar amount of flattening in K1 and K2 after CCL, which maintained the same difference between the two values. K apex value is more commonly evaluated after CCL, and significant decreases in K apex are known to occur.^27,28,29,30^ In our study, we observed a significant decrease in K apex values at postoperative month 6. When evaluating the efficacy of CCL, reduction or stability in K apex values after 1 year is an important indicator in the follow-up of keratoconus patients.

In the present study, TCT measured by Sirius topography showed a significant decrease at postoperative month 6, then increased steadily until year 5. However, despite the increase after 6 months, the decrease was significant compared to preoperative values. TCT only returned to preoperative values after 5 years. Sarac et al.^31^ performed CCL using mechanic or phototherapeutic keratectomy and observed significant decreases in TCT at postoperative 3 years in both groups. Wittig-Silva et al.^32^ performed CCL using a modified Dresden Protocol and reported that at 3-year follow-up, TCT measured by ultrasonic pachymetry was significant reduced in the control group and unchanged in the CCL group, whereas TCT measured using Orbscan computerized videokeratography showed significant decreases, consistent with our study. In another study, a significant decrease was observed in corneal thickness measured using Pentacam at 1 year after CCL.^33^ Postoperative corneal haze leads to erroneous results when measuring corneal thickness using optical methods. There are studies showing that there is no significant change in corneal thickness measured using both Orbscan computerized videokeratography and ultrasonic pachymetry in long-term follow-up due to the reduction in corneal haze.^23,32^ However, in these studies, TCT measured using Orbscan computerized videokeratography reached preoperative values after only 3 years of follow-up, TCT took longer to reach preoperative values in our study, despite the reduction in haze observed 2 years after CCL treatment. The effect of CCL on corneal thickness has not yet been fully explained. Many factors have been proposed as causes of this effect, such as stromal structure, postoperative dehydration, and epithelial recovery and distribution.^34,35^ Moreover, measurement techniques used in the early postoperative period may give inaccurate results due to artifacts and haze.

There are also studies that have investigated how CCL affects optical quality.^36,37,38^ In our study, we found that the corneal aberration parameters of HORMS and spheric aberration decreased significantly and BCVA increased after CCL. Kosekahya et al.^33^ observed significant decreases in total RMS, HORMS, vertical coma, and spherical aberration values in Pentacam measurements of corneal aberrations and a significant increase in BCVA at 1 year after CCL. In contrast, Wisse et al.^37^ observed no changes in total HORMS, coma, and trefoil aberrations in the 1-year follow up of 164 patients, while there was a significant decrease in spherical aberrations and a significant increase in BCVA.In our study of 5-year follow-up after CCL, we found that spherical aberrations decreased after year 3 and that BCVA also improved in parallel.

### Study Limitations

The limitations of this study are its retrospective design, the need for a larger patient population, and the lack of ultrasonic pachymetry measurements.

## Conclusion

In conclusion, based on the clinical and topographical data from 5-year follow-up of therapeutic CCL in patients with progressive keratoconus, it was determined that CCL prevents disease progression by 95%, increases vision, decreases higher-order aberrations and spherical aberrations, and can now eliminate the need for corneal transplantation in most cases. For all of these reasons, the long-term outcomes of CCL in progressive keratoconus are highly satisfactory, and CCL continues to be the first choice of treatment for patients with progressive keratoconus.

## Figures and Tables

**Table 1:**
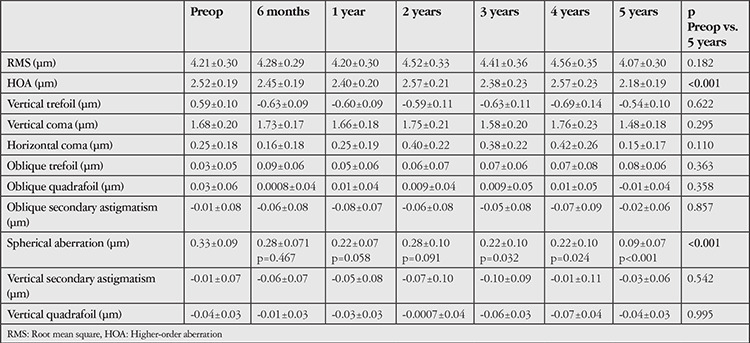
The patients’ preoperative (preop) and postoperative mean corneal aberration values
